# RNAStructuromeDB: A genome-wide database for RNA structural inference

**DOI:** 10.1038/s41598-017-17510-y

**Published:** 2017-12-08

**Authors:** Ryan J. Andrews, Levi Baber, Walter N. Moss

**Affiliations:** 1 Roy J. Carver Department of Biochemistry, Biophysics, and Molecular Biology, Iowa State University, 2437 Pammel Drive, Ames, IA 50011 USA; 2 Biology Information Technology, Iowa State University, 2310 Pammel Drive, Ames, IA 50011 USA

## Abstract

RNA plays important roles in almost every aspect of biology, and every aspect of RNA biology is influenced by its folding. This is a particularly important consideration in the era of high-throughput sequencing, when the discovery of novel transcripts far outpaces our knowledge of their functions. To gain a comprehensive picture of biology requires a structural framework for making functional inferences on RNA. To this end we have developed the RNA Structurome Database (https://structurome.bb.iastate.edu), a comprehensive repository of RNA secondary structural information that spans the entire human genome. Here, we compile folding information for every base pair of the genome that may be transcribed: coding, noncoding, and intergenic regions, as well as repetitive elements, telomeres, etc. This was done by fragmenting the GRCh38 reference genome into 154,414,320 overlapping sequence fragments and, for each fragment, calculating a set of metrics based on the sequence’s folding properties. These data will facilitate a wide array of investigations: e.g. discovery of structured regulatory elements in differential gene expression data or noncoding RNA discovery, as well as allow genome-scale analyses of RNA folding.

## Introduction

Once thought to be solely an intermediary between the genome and proteome, RNA is now known to be a key player in the biology of all living things (as well as viruses, viroids and transposable elements). In addition to carrying the genetic information needed to generate proteins, RNA can also act as a catalyst^1,2^, encode signals for subcellular localization^3–5^, and regulate gene expression^6^. RNA’s gene regulatory roles can occur in-*cis*, via sequence/structure elements embedded within messenger (m)RNAs: e.g. regulatory elements in untranslated regions (UTRs) and introns can affect translation^5,7^ alternative splicing^8^, and mRNA lifetime/abundance^9^ Additionally, RNA can regulate gene expression in-*trans* via intermolecular base pairing between mRNAs and noncoding (nc)RNAs such as micro (mi)RNAs^10,11^, as well as through interactions that mediate the epigenetic control of gene expression: e.g. the *Xist* long (l)ncRNA that associates with one of two X chromosomes in mammalian females and leads to chromosomal condensation and inactivation. A great deal of work has gone into identifying, understanding, and archiving cis- and trans-regulatory sites on human mRNAs. For example, the regulatory (reg)RNA^12^ web server contains a large repository of data on various motifs, such as splicing regulatory motifs, polyadenylation signals, and mRNA degradation elements; drawing from additional databases of cis-regulatory elements: e.g. ERPIN^13^, fRNAdb^14^, and Rfam^15^. The RBPMap web tool allows users to deduce primary sequence binding motifs on RNAs for a wide array of regulatory proteins^16^.

The list of new ncRNAs and novel functions of ncRNAs grows daily: however, this is likely only the “tip of the iceberg”. The current release of the human genome reference sequence (GRCh38.p10) is 3,088,269,832 base pairs (bp) long (Genome Reference Consortium). Approximately 90% of our genome is transcribed into RNA^17^ yet, only 1.5% encodes protein. This results in a staggering amount of potentially functional RNA to be characterized. Although some of this pervasively transcribed RNA may be “junk”, many of these sequences are differentially expressed (compiled within the NRED database^18^) in diseases such as cancer^19–21^, or under conditions of cellular stress^22^. Considerable effort is underway to identify and elucidate the functions of ncRNAs. A number of labs have been recording and annotating sequences recovered from high-throughput sequencing and more traditional molecular and cell biology approaches. Collections of ncRNA sequences are being built into databases such as Rfam^15,23^, lncRNAdb^24,25^, LNCipedia^26,27^, mirBASE^28–32^ and RNAcentral^33–35^. These important projects are compiling well-annotated and, in many cases, functionally validated ncRNAs alongside other valuable data. The RNA families (Rfam) database, for example, contains entries for families of ncRNAs linked by homology. Rfam entries contains information describing ncRNA biosynthesis, localization, phylogenetic distribution and functional roles, as well as evolutionary conservation of primary sequence and, importantly, secondary structure. Conservation of secondary structure is a defining feature of ncRNAs and is used in essentially all ncRNA prediction programs^36^.

A powerful, and popular, program for genome-wide ncRNA prediction is RNAz^37–40^. This program is based on a support vector machine (SVM) that is trained on data from known ncRNAs contained within Rfam. Two primary training parameters are used for ncRNA classification: a structure conservation index (SCI), which measures conservation of secondary structure and a thermodynamic z-score, which measures the propensity of a particular sequence to form a defined and energetically stable structure. Typical ncRNAs have structures with high conservation and propensity to form structure. RNAz was previously used to scan through whole genome alignments comparing human vs. animal genomes (ranging in similarity down to zebrafish) to identify putative ncRNAs. This yielded >30,000 high-confidence predictions with ~1,000 that were conserved throughout all vertebrates^41^. The identification of so many deeply conserved structured RNAs highlights their likely ubiquity and importance.

In both coding and noncoding RNAs, secondary structure plays key roles throughout their functions. The diversity of RNA functions and potential for physiological impact (much like with proteins) is made possible by the ability of RNA to fold into unique functional structures. Functional RNA structures form thermodynamically stable base pairs that have been selected for by evolution^42^. This is the key principle behind the thermodynamic z-score implemented in RNAz: functional RNAs have a more stable folding energy than randomized sequences. Specific folds can be recognized by regulatory proteins^43^, occlude/present functional motifs^44^, or alter the distance between functional sites^45^.The impact of RNA structure on alternative splicing, for example, has been particularly well-studied^46,47^. Additionally, awareness of the importance of non-specific RNA structure (e.g. regions that do not form particular folds or adopt dynamic structures) is growing. For example, thermodynamically stable regions within open reading frames are proposed to modulate the speed of translation and thus affect protein folding^48,49^. RNA intramolecular thermodynamic stability is important in mediating its accessibility for intermolecular interactions: e.g. stable regions in UTRs are less accessible to miRNA binding, thus affecting miRNA-mediated gene silencing^50^. Dynamic RNA structure also has significance to disease: single nucleotide polymorphisms (SNPs) can affect RNA folding in ways that impede healthy function by disrupting specific motifs or altering conformational equilibria^51,52^.

Advances in sequencing technology will continue to massively expand the list of interesting RNA sequences and, excitingly, also provide information on secondary structure. Several *in-vivo* RNA structure probing techniques have been developed to acquire transcriptome-wide folding information^53^. Snapshots of the human “RNA structurome” (ranging across tissue/cell types, disease states, and treatments with drugs) will become more common in the near future; informing our knowledge of human biology and advancing our understanding of pathogenesis. For this reason, and the reasons discussed above, it is critical to have a knowledge framework in place to understand the roles of RNA structure in human biology. This is the motivation for the creation of the RNAStructuromeDB. Here, we have compiled computed RNA folding information across the entire human genome, irrespective of whether or not it is known to be transcribed. The RNAStructuromeDB is a web-accessible (https://structurome.bb.iastate.edu) repository for investigators to obtain structural metrics for any RNA sequence originating from the human genome. To further aid investigators we have put the data into context by incorporating comprehensive Gencode annotations^54^ using the biological database schema Chado^55^. This allows the rapid comparison of differential gene expression data (e.g. to identify regulatory RNA structures) or transcriptome-wide RNA biochemical probing data against the RNAStructuromeDB facilitating these, and other types of studies.

## Results and Discussion

The RNAStructuromeDB holds the results of a genome-wide computational analysis in which we folded the entire human genome. The results of this analysis are comprised of folding metrics which indicate every region of the genome’s propensity to generate structured RNA molecules. Tools have been built by which users can quickly download these metrics or use them to search for novel RNA structural elements or to assess the global folding properties of a transcript. Here we present the metrics that will aid in the investigation of RNA structure/function, and the tools which have been developed to view these metrics effectively.

### Data types

To analyze a genome as large as the human genome requires fragmenting the sequence data into smaller pieces that are both computationally tractable, as well as biologically meaningful. The fragmentation approach taken in the construction of the RNAStructuromeDB is similar to those that were successfully used for the analysis of RNA structure in the human and other genomes^39–41,56–58^ as well as in specific lncRNAs^59^. Here we consider discrete, overlapping windows whose size was selected based on several considerations. Due to the kinetics of RNA folding, RNAs typically fold into structures composed of smaller motifs; for example, lncRNAs like HOTAIR (2,421 nt) are comprised of locally folding domains (4 domains), each containing many local substructures (e.g. RNA hairpins^60^). Additionally, the majority of known RNA sequences/structures archived in Rfam are short (<200 nt long). Incidentally, a window size spanning 100 to 150 nt was shown to be most accurate at predicting base pairs in known cis-regulatory structures of large mRNA molecules (>85,000 nts long)^61^. Finally, folding algorithms scale O(N^3^) in time and O(N^2^) in memory, where N is the sequence length^62^; thus, shorter sequences, in addition to yielding more accurate predictions, can be folded more quickly and efficiently. With these factors in mind, a window size of 120 nt was selected to maximize the chance of encapsulating structured elements, while also optimizing prediction accuracy and computational resources. A step size of 40 nt was selected to ensure the entire genome is canvassed with multiple frames, increasing the likelihood of capturing all structured elements. This window and step size resulted in the generation of 154,414,320 windows which were analyzed in both strand orientations.

For each window, five folding metrics were calculated and archived. The Gibb’s minimum free energy (MFE) of folding (ΔG), which estimates the thermodynamic stability of the most stable 2D conformation of an RNA given Turner nearest neighbor energy parameters^63,64^ (a set of experimentally measured values taken from the analysis of many small RNA motifs). The ΔG was calculated using the program RNAfold, which is a component of the ViennaRNA package^63^; predictions were made at 37 °C (human body temperature) and values are reported in kcal/mol. 2D structures associated with window MFE predictions were captured and archived in “dot-bracket" notation; here, paired nt are represented by matched brackets “()” and single stranded nt by dots “.”. To determine if the MFE ΔG depends on the nt *order* or *composition*, we compared the native sequence ΔG vs. *in silico* randomized sequences with the same nt composition. In general, structured ncRNA molecules have lower (more stable) MFE values than random sequences of the same nucleotide composition^65^; a property which can be exploited for structured RNA motif discovery^66^. For each window, we report the thermodynamic z-score. The z-score is calculated by taking the difference between native MFE (*ΔG*_*native*_) and the mean MFE of random sequences ($\overline{\Delta {G}_{random}})$, then normalizing by the standard deviation, as described in equation (1) in the Materials & Methods section. The z-score sign indicates if the native MFE is either lower (negative) or higher (positive) than $\overline{\Delta {G}_{random}}$ and its magnitude indicates the standard deviations *ΔG*_*native*_ is from $\overline{\Delta {G}_{random}}$. A z-score less than −1, for example, indicates a *ΔG*_*native*_ which is *one standard deviation lower* than random. Negative z-score suggests that a sequence’s *order* was selected by evolution to fold into a stable structure^42^: e.g. if the order is important, shuffling nt will lead to less stable folds by disrupting native (evolved) pairing contacts that give the RNA thermodynamic stability. Positive z-scores are harder to interpret, however, they could suggest regions of RNAs that are evolved to be more accessible/unstructured^59^. The z-score can also be qualified using the p-value, which is calculated as the fraction of *ΔG*_*random*_ values with more thermodynamic stability than *ΔG*_*native*_. This provides a measure of the z-score quality, where p-values close to zero indicate higher prediction confidence: few or no randomized sequences are more stable than the native sequence; this also indicates if the randomization number is high enough to yield reliable z-scores.

Two values are recorded which measure statistical properties of the RNA 2D folding ensemble: the ensemble diversity (ED) and the frequency of the MFE (fMFE) metrics. Structured RNAs have rough folding landscapes (with many “suboptimal” folds that are near in energy to the native MFE fold) and, in some cases, are capable of occupying several conformations. The probably of finding any of these structures within the Boltzmann ensemble is mathematically described by the partition function^67^, which was calculated using RNAfold^63^. From this partition the ED is calculated by taking the average base-pair “distance” between all structures in the ensemble, where distance is defined as the number of base pairs different between structures^65^. The ED then, is a metric to mathematically signify the *variety* of folding structures within the ensemble (a low ED implies a small number of similar structures are present, while a high ED suggests several alternative folds or a lack of overall structure in the ensemble^68^). The fMFE metric is the probability of finding the MFE within the Boltzmann distribution of structures, where a high probability suggests the MFE structure is more likely to be the dominant fold.

The five archived metrics each suggest regions of the human genome that may generate RNAs with interesting folding properties. The MFE ΔG defines the thermodynamic stability, while the z-score suggests whether or not that stability is unusual (given the sequence composition). A region can have a very stable ΔG, but mediocre (or positive) z-scores because the order is unimportant vs., for example, the GC content. The p-value estimates the quality of the z-score. If almost every randomized sequence is less stable than native, then the likelihood of the order being significant is higher. The ED and fMFE indicate the diversity of the RNA folding ensemble in a region and how well-represented the MFE prediction is in the 2D structural ensemble, respectively. These can suggest which regions have well-defined folds (e.g. with low ED and high fMFE) or where folding may be diverse (e.g. dynamic regions or regulatory structural switches that have higher ED and low fMFE). Taken together, these metrics can also suggest which regions may be harboring functional structures. Overlapping windows with interesting folding metrics can be concatenated to define larger regions of interest, which can then be used for comparative sequence/structure modeling^69^ or ncRNA prediction (e.g. using approaches such as RNAz^37–40^. Examples of how the metrics contained in the RNAStructuromeDB can be used are discussed below in the “Examples” subsections.

### Data accessibility

There are three main methods for accessing the data: flat files, data tables, and via an interactive genome browser. Users can freely download raw data as flat files in generic feature format version three (GFF3) directly from the download page (https://structurome.bb.iastate.edu/downloads). GFF3 is a standardized file format developed by The Sequence Ontology^70^ for the sharing of genomic information in a unified manner and is compatible with several bioinformatics programs. This file contains each window’s sequence, coordinates, strand orientation (forward or reverse), MFE, z-score, p-value, ED, fMFE and dot-bracket 2D structure. This data format will allow interested end-users to parse out trends using their preferred methods or protocols; however, users may want to filter the metrics, downloading only information relevant to their needs/interests. Therefore, we have also created an interface where a user can filter and search for data based on any combination of genomic coordinates, Ensembl IDs, gene symbols, or folding metrics—facilitating varied inquiries. Investigators interested in multiple genes can quickly search for, populate, and download folding metrics coinciding with each gene as a single consolidated CSV (comma-separated values) file. Conversely, a user may have discovered an RNA molecule lacking any established annotations; as long as coordinates are supplied, this user will find folding metrics for their novel transcript as well. This genome-wide analysis provides the freedom to obtain data from any known or novel transcript.

The JBrowse genome browser provides significant insights into the structural landscape of each chromosome via a customizable visual interface. Within the JBrowse interface, folding metrics have been adapted to allow for rapid structural inferences to be made: they are displayed as bar graphs canvasing each chromosome sequence. Each folding metric (MFE, z-score, p-value, ED, and fMFE) has been split into separate graphical tracks that can be displayed parallel to genomic annotations. A user interested in a specific transcript will be able to observe the folding metrics of its constitutive features (exons, introns, and UTRs are all displayed with unique visualizations), while at the same time be able to see the folding metrics of the entire gene within greater genomic context. The human eye is very good at identifying patterns (e.g. within the structure of the data represented in the JBrowse tracks) and visualizing the genome, transcriptome and predicted RNA structurome simultaneously can facilitate discovery. Examples are given below.

### Example 1: The MALAT1 lncRNA

Currently, thousands of lncRNAs have been discovered in the human transcriptome and a growing list has been assigned functions and are implicated in diseases such as cancer^71–73^; however, most lncRNAs and their functions remain uncharacterized^73^. One aspect of lncRNA function that remains controversial, is the role played by RNA secondary structure. Many reports describe extensive local or global folding for lncRNAs^59,60,74^; yet, statistical evidence of structure conservation remains tenuous^75^. It is worth noting that, even in the absence of high structural conservation, RNA folding can play functional roles^76^. Analyzing lncRNA folding landscapes and building 2D structural models of these molecules will aid in understanding their mechanisms of action (e.g. in detecting regulatory structural motifs) and, possibly, in developing therapeutic strategies^77^ to modulate function. lncRNAs tend be modular in structure, consisting of multiple structured domains^78^. Sliding window approaches for RNA structure detection are able to roughly define the extent of these domains^59^. The RNAStructuromeDB suggests these domains for all potential human lncRNAs with the folding metrics and local structural models necessary to begin to decipher the structure and function of these transcripts.

**Figure 1:**
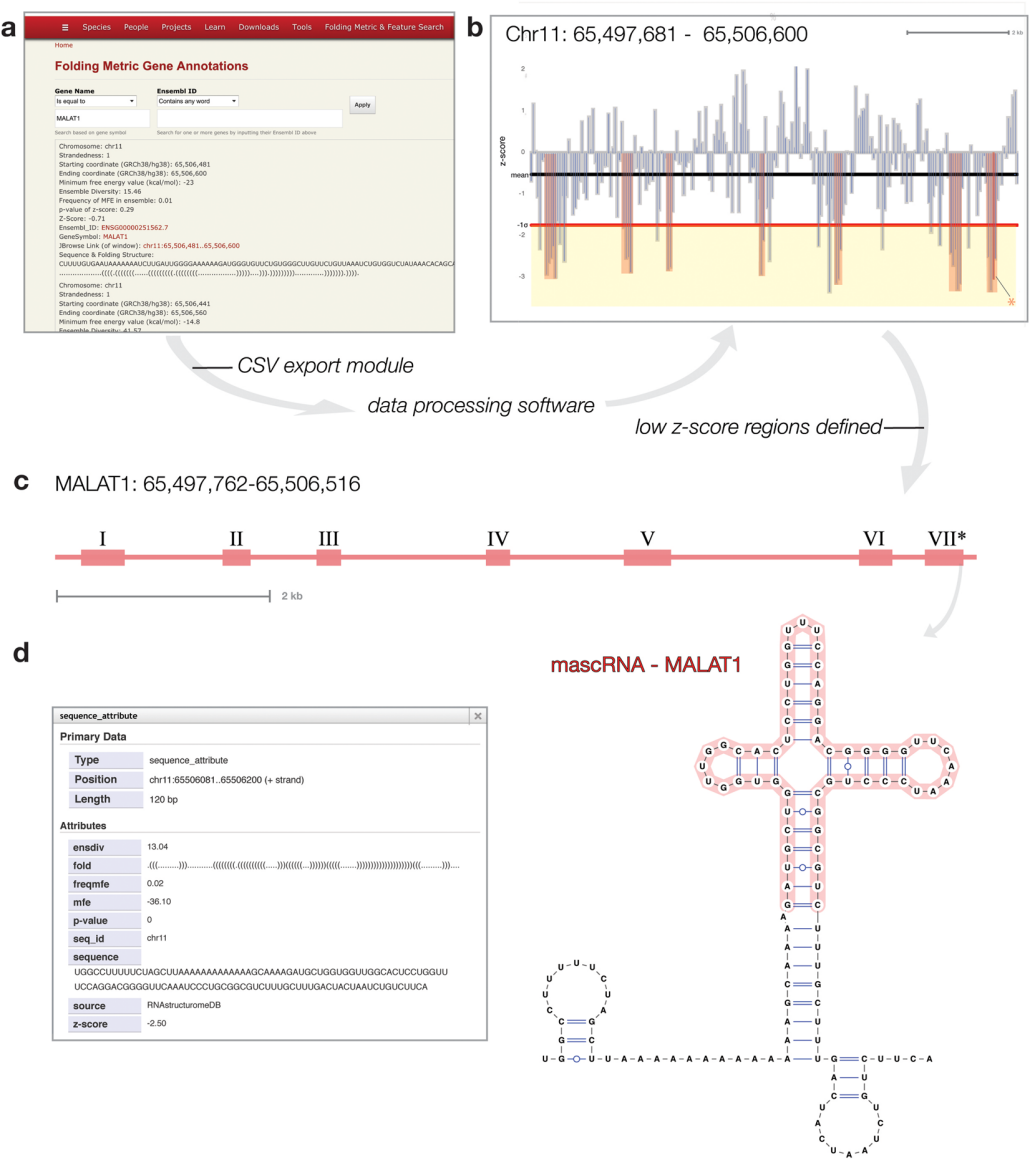
Pipeline for defining “structural regions of interest” in MALAT1 lncRNA. (**a**) Folding metrics can be obtained by inputting a desired target in a data table interface and downloading a corresponding CSV file from a link at the bottom of the page. (**b**) Here, the CSV file is analyzed in excel to find windows with one standard deviation lower than average z-scores; implying their likelihood to contain structured RNA sequences. (**c**) These windows were concatenated to define larger regions of interest within the MALAT1 lncRNA (labeled I-VII). (**d**) One of these regions (VII*) contains a window which encompasses the mascRNA of MALAT1. The JBrowse popup of this window is shown; directly adjacent is the window’s sequence and MFE dot-bracket structure as visualized in VARNA (with annotation highlighting the mascRNA sequence shown in red).

For example, MALAT1 (metastasis-associated lung adenocarcinoma transcript 1) is a highly conserved (throughout 20 mammalian species, including mouse and human^79^) lncRNA, which is involved in numerous cellular processes (e.g. transcriptional regulation^80^, alternative splicing^81^, and cellular localization^82^) and implicated in disease states: such as cancer^83^ and diabetes^84^. Here, the RNAStructuromeDB data table interface is used to define the structured domains of MALAT1 (summarized in Fig. 1). Upon inputting the MALAT1 target (specified by genomic coordinates, gene symbol, or Ensembl ID), all overlapping folding metric windows are extracted from the DB. These can be browsed on the website or downloaded as a single CSV file. With these data, investigators can use any method to define structured regions; in this example, regions with overlapping windows having z-scores 1 σ more negative (< −1.70) than the average MALAT1 z-score (−0.51) were concatenated into domains (similar to previous work on the Xist lncRNA^59^). This resulted in seven domains (labeled I–VII in Table 1) likely to generate structured RNAs and 11 individual windows with z-scores less than −1.70. Concatenated domains range from 160 to 280 nt (comprising two to six overlapping windows). Interestingly, in addition to their low z-scores, the defined domains also have lower than average *ΔG*_*native*_ (in all but domain II) and ED scores (Table 1), suggesting stable folding with one (or few) dominant conformations in the structural ensemble. Structure models for individual windows can be analyzed directly from the RNAStructuromeDB. For example, domain VII contains a window (positions chr11:65,506,081–65,506,200) that contains a known structured element important to MALAT1 maturation and that also has independent functions in the cell: the MALAT1-associated small cytoplasmic (masc)RNA^85^. The terminal window in this domain contains the predicted model of the mascRNA (highlighted in Fig. 1d), which correctly predicts the mascRNA tRNA-like folding this is essential to its function. Sequences corresponding to each longer domain can be used for structure modeling: e.g. using RNAfold locally or through the RNAfold web server^86^, be used for BLAST^87^ searches to identify homologs, aligned to related sequences, and used for consensus folding, comparative sequence/structure analyses and ncRNA prediction (e.g. using the RNAz server^38–40^). Links to all of these tools can be found on the RNAStructuromeDB website.

**Table 1:** Low z-score windows and regions of MALAT1.

region	Position		z-score		(Δ*G*_*native*_)		ED	
	start	end	average	minimum	average	minimum	average	minimum
—	65497841	65497960	**—**	−2.27	**—**	−47.4	**—**	18.51
I	65497961	65498240	−2.61	−2.94	−34.34	−42.7	14.24	5
—	65498201	65498320	**—**	−2.14	**—**	−32.6	**—**	16.36
—	65498281	65498400	**—**	−2.56	**—**	−53.9	**—**	7.34
II	65499361	65499600	−2.38	−2.49	−24.42	−25.9	11.37	8.23
—	65499961	65500080	**—**	−2.06	**—**	−21	**—**	12.38
III	65500161	65500320	−2.66	−2.7	−24.76	−26.2	12.08	12
IV	65501841	65502000	−2.57	−2.86	−28.26	−35.5	11.84	4.25
—	65502001	65502120	**—**	−2.54	**—**	−37.5	**—**	19.23
—	65502881	65503000	**—**	−2.2	**—**	−18	**—**	12.84
—	65503081	65503200	**—**	−3.28	**—**	−40.1	**—**	5.03
V	65503201	65503400	−2.72	−3.07	−33.49	−38.1	12.56	10.02
—	65504041	65504160	**—**	−2.26	**—**	−35	**—**	14.03
—	65504921	65505040	**—**	−1.87	**—**	−31.1	**—**	13.72
—	65505081	65505200	**—**	−2.17	**—**	−38.5	**—**	12.31
VI	65505281	65505560	−2.72	−3.08	−37.26	−44.3	21.44	17.56
—	65505601	65505720	**—**	−2.23	**—**	−38.4	**—**	7.1
VII*	65505961	65506200	−2.79	−3.25	−34.45	−36.1	16.02	12.72
full	**65497681**	**65506600**	**−0.51**	**−3.28**	**−24.52**	**−59.6**	**24.99**	**4.25**

Each region was defined as a low z-score region if it contained two or more consecutive windows with z-scores 1 σ lower (< −1.71) than the MALAT1 average (−0.50). Also reported is each region’s minimum and average *ΔG*_*native*_ (kcal/mol) and ensemble diversity (ED) value. Single windows with low z-scores (< −1.71) are also shown. Region names (I-VII) correspond to those shown in Fig. 1.

### Example 2: The VEGFA gene

**Figure 2:**
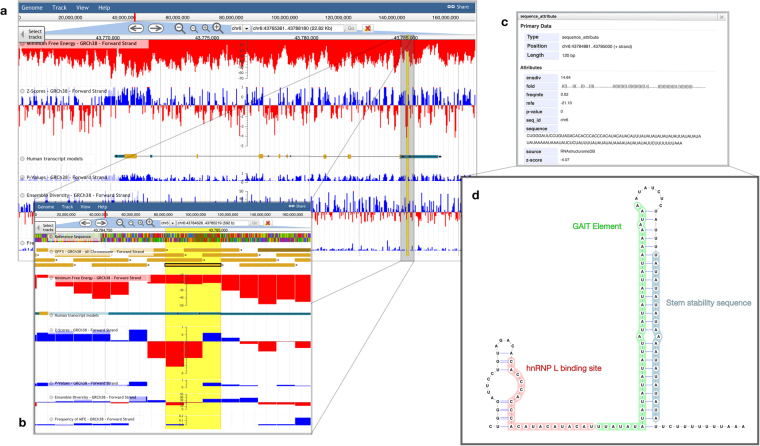
JBrowse target identification pipeline. **(a)** The general JBrowse interface is shown with all five forward strand metrics shown as bigWig tracks. The z-score and ensemble diversity tracks have been set to pivot in color around their global mean values. The “Human transcript models” track visualizes transcripts from the Gencode comprehensive set (v26) showing UTRs in blue, exons in yellow, and introns in black. Here we have collapsed all transcripts in the area into a single entity via the JBrowse user interface. **(b)** The lowest z-score window of the region has been highlighted and focused on to identify and select the corresponding window (yellow rectangle which we have outlined with a thick black border). **(c)** Upon clicking this window in JBrowse, a pop-up is generated which reports all corresponding metrics. **(d)** The sequence and MFE dot-bracket structure were viewed and annotated in VARNA to show where the predicted structure overlaps with the previously described VEGFA riboswitch. Functional sites are color annotated and labeled.

In 2008, the first human riboswitch was discovered in the mRNA of vascular endothelial growth factor-A (VEGFA)^44^. Within this mRNA, is a region of the 3′ UTR which can adopt two structural conformations, each of which leads to different translational levels of VEGFA. The adoption of either conformation is mediated by protein binding which occurs as a result of hypoxic signaling. When expressed in high abundance (under hypoxic conditions) the regulatory protein hnRNP L binds to the VEGFA riboswitch causing two sequences, known as the GAIT (gamma interferon inhibitor of translation) element and the stem stability sequence (Fig. 2d), to anneal to each other: inhibiting association with the GAIT complex and stimulating VEGFA expression. When hnRNP L is not highly expressed (under normoxic conditions), these elements form their own discrete hairpins (Fig. 2d) and the GAIT element is able to bind the GAIT complex repressing VEGFA expression.

Figure 2 shows the basic pipeline involved in using the genome browser. Upon opening the JBrowse tool, a user is able to input genomic coordinates or Ensembl ID and navigate to their region of interest. In the case of VEGFA, the Ensembl ID (ENSG00000112715.21) was entered to navigate to the genome coordinates. Graphical representations of folding metrics are displayed as tracks, which flank the VEGFA transcript model (Fig. 2a). Transcript models display exons (yellow), introns (black line), and UTRs (blue) as separate entities, allowing for quick visual analyses. VEGFA has a multitude of low z-score windows (197 windows below average), the majority of which (85%) lie within introns and UTRs. The lowest z-score window in the VEGFA gene resides in the 3′ UTR; excitingly, this window fully encompasses the VEGFA riboswitch, highlighting the utility of the metrics archived in the RNAStructuromeDB as well as the ease of picking out regions of interest visually using JBrowse. Users can highlight this region to “zoom in” to investigate that particular window (Fig. 2b).

Individual windows can be selected to view a “pop-up” window (Fig. 2c) containing: folding metrics, RNA sequence, and a dot-bracket structure model (dot-bracket structures can be conveniently represented in 2D using the VARNA java applet^88^). Using VARNA, we annotated the 2D image with the key riboswitch elements (Fig. 2d). The model MFE structure archived in the RNAStructuromeDB recapitulates the translation permissive (TP) conformation that dominates under hypoxia, where the GAIT element and stem stability sequence are annealed to each other. The differences in the MFE model and the TP conformation described in the literature occur primarily at the hnRNP L binding site. The literature model is based on enzymatic probing of an *in vitro* generated construct that terminates 5 nt upstream of the hnRNP L interaction site. The MFE model, however, includes 13 additional upstream nt because of the window size used. These additional nt allow for the formation of a short hairpin stem that sequesters part of the hnRNP L binding site in a short hairpin loop (Fig. 2d). It is worth noting that the *in vitro* model for the TP conformer is poorly defined in this region: high reactivity spans the 5′ end and a strong cleavage site occurs within one of the proposed helices in this model^44^. The MFE model posits novel interactions of the hnRNP L binding nt that may be biologically significant.

### Example 3: Hyperedited regions

In addition to the most well-known RNA post-transcriptional modifications (splicing, capping, polyadenylation, tRNA base modifications, etc.) RNA molecules can undergo endogenous enzyme-mediated chemical modifications; a process known as RNA editing^89^. The most common editing event in the human cell, is that performed by the adenosine deaminase acting on RNA (ADAR) enzyme family: Adenosine to Inosine (A-to-I) deamination. These editing events are ubiquitous throughout the body^90^ and likely play a role in regulating gene expression^91^ and establishing disease states^92^. Inosine is recognized by most cellular machinery (as well as the enzymes used in RNA sequencing) as guanosine; indeed it is the A-to-G transitions observed as variants in sequencing reads that allows one to quantify the percent A-to-I editing at a nt^93^. Efforts towards creating an “inosinome Atlas” (by conducting whole genome and transcriptome sequencing of human cells to detect A-to-I editing events) resulted in the identification of ~3 million A-to-I editing sites^90^. Given their quantity, likely roles in regulating gene expression, and their implication in disease states it is important to gain insight into the structural features of A-to-I “hot-spots”.

**Figure 3:**
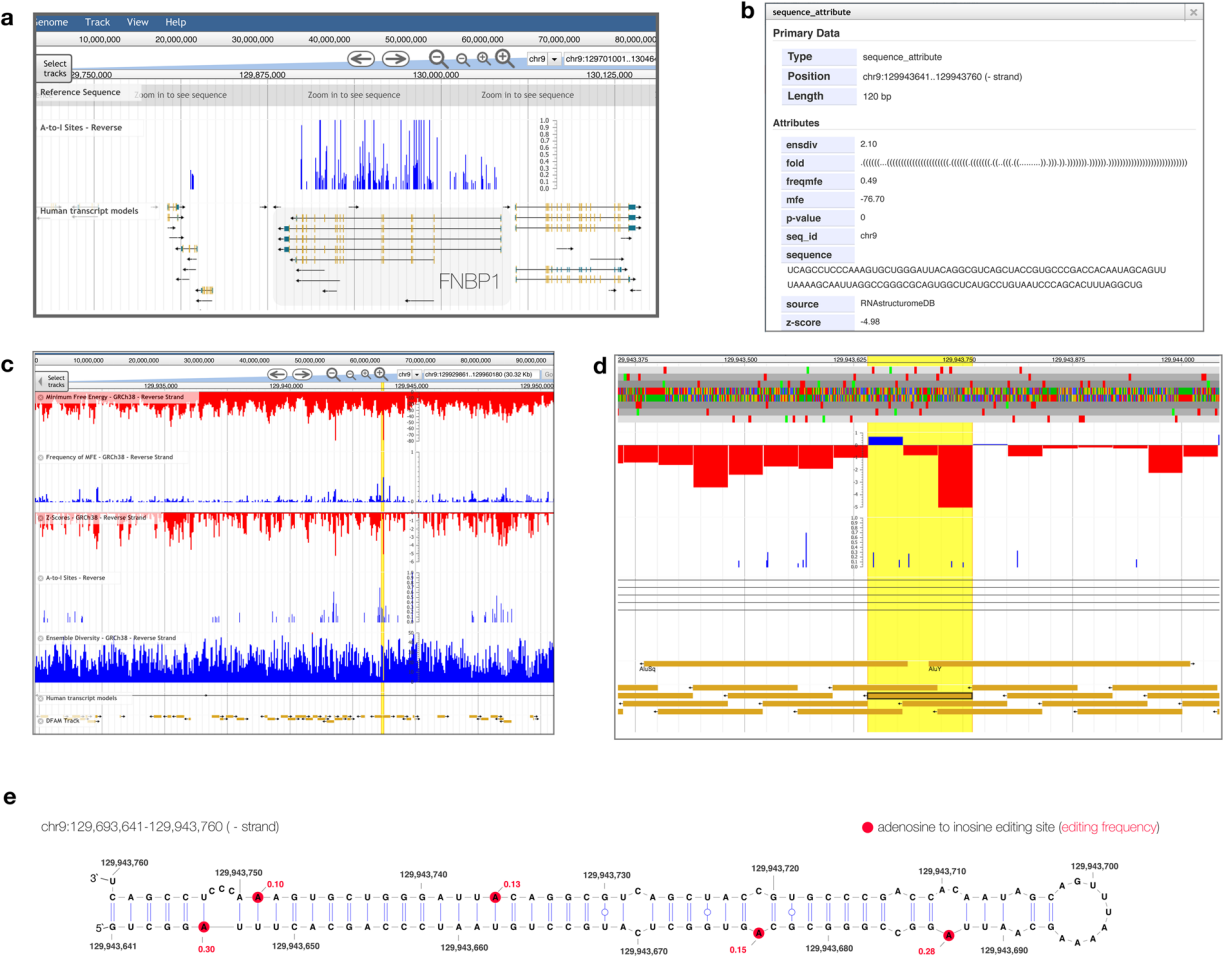
Mapping A-to-I editing events in the FNBP1 transcript. **(a)** A-to-I editing event frequencies (0 to 1) observed in ref.^88^ were converted to bigWig tracks and added to JBrowse via the native user interface; editing frequencies are shown (in-blue) directly above nine transcript models of FNBP1 from Gencode annotations (v26). Transcript model models depict individual transcripts with their UTRs in blue, exons in yellow, and introns as black lines. **(b)** A detailed pop-up of an interesting window located within this region is pictured. **(c)** This window then is highlighted in yellow and shown in greater detail using JBrowse. (**d)** Further zooming towards this window (yellow rectangle outlined in black) we can see that it overlaps five A-to-I editing sites, and spans the region between two inverted Alu elements. (**e)** The folding structure of this window is shown with its A-to-I editing sites and editing frequencies shown in red.

ADAR enzymes have a strong preference for double stranded (ds)RNA regions^94^. ADAR editing was first described in helical regions of very long hairpins found in 3′ UTRs^95,96^ Subsequently, many editing sites were found; for example, within regions containing Alu elements^97^ whose inverted repeat structure facilitates hairpin formation. This structure-preference for ADAR makes the folding metrics within the RNAStructuromeDB particularly useful for interpreting patterns of A-to-I editing data. We utilized the customizability of JBrowse to visualize A-to-I editing sites alongside folding metrics in order to examine the structural landscape corresponding to edited regions. We prepared tracks using data from a study which looked at RNA editing in human B cells^91^ where several transcripts were discovered to be hyperedited. For example, the formin binding protein 1 (FNBP1) transcript was found to have the most editing events (291) of any gene, and when viewed alongside folding metrics it was clear that predicted structured regions aligned well to editing sites (Fig. 3). A particularly striking example is the window with metrics shown in Fig. 3b (as well as the genome browser tracks shown in Fig. 3c and d); this window has not only a highly-negative z-score (almost five standard deviations more stable than random), but low (favorable) MFE, ED and high fMFE values as well. This window overlaps a cluster of five highly-edited sites, which are annotated on the predicted structure shown in Fig. 3e. Interestingly, in addition to the highly-stable hairpin in the individual window, concatenating adjacent windows which overlap editing sites (or are within 40 nt and have a less than average z-score) can define a larger hairpin structure (677 nt long) where this particular window forms the terminal hairpin stem loop (Figure S1). Nine out of the 15 windows overlapping this region had z-scores lower than the transcript average (Supplementary Table 1) showing how the sliding window approach can still be used to define domains > the window size used (120 nt in this case). In this particular region there have been two inverted Alu element insertions (Fig. 3d), which provide the complementarity for forming such a large stem structure. As this whole region is transcribed as part of an intron, it is possible for it to fold as predicted into the long hairpin, which is structurally similar and similar in length to the long 3′ UTR hairpins described in earlier studies of ADAR editing^95,96^.

In addition to helping define the extent of structured domains recognized by ADAR (which could facilitate the design of assays to study editing, by removing/adding domains to reporters, etc.) the information in the RNAStructuromeDB can also offer insights into the detailed structural contexts of A-to-I editing sites. The primary sequence context of these editing sites is being investigated, with insights being made into flanking nucleotide preferences^90^ and tools have been created that predict editing sites based on these primary sequence rules^98^; the RNAStructuromeDB may enhance these investigations by providing secondary structure context. For example, we can compare the percent editing at an individual site to its structural context. In Fig. 3e, three editing sites occur in AU pairs (two flanked by Watson-Crick pairs, and the other at the end of a helix upstream of a 3 nt bulge-loop) and two occur in internal loops opposite to cytosine residues. In all cases the inosine substitution would be expected to minimally disrupt folding energy (AU to IU pairs) or enhance stability (AC mismatch to IC pair). Additionally, the flanking nearest-neighbor nt can also affect stability. Thus, the folding information in the RNAStructuromeDB might be able to help discern sequence/structural features of editing hot spots that can help predict sites of editing as well as the effect of editing on RNA folding.

## Conclusion

The RNAStructuromeDB is a repository of useful RNA folding metrics and a powerful vehicle for exploring the human genome via RNA structure. It allows users to browse, access, and retrieve the data quickly and flexibly, which will facilitate a wide array of researches. We presented three examples of how this database can be used: to generate a map of RNA folding throughout the MALAT1 lncRNA, VEGFA gene, and FNBP1 transcript. In each example, global properties of folding (e.g. the enrichment for stable folding in intronic or UTR regions), as well as the identification of functional motifs (e.g. the identification and modeling of the VEGFA riboswitch and stable hairpin in FNBP1) could be quickly deduced. We anticipate that this tool will have many applications both in basic research and in the therapeutic targeting of disease-associated human RNAs.

## Materials and Methods

### Overview

**Figure 4:**
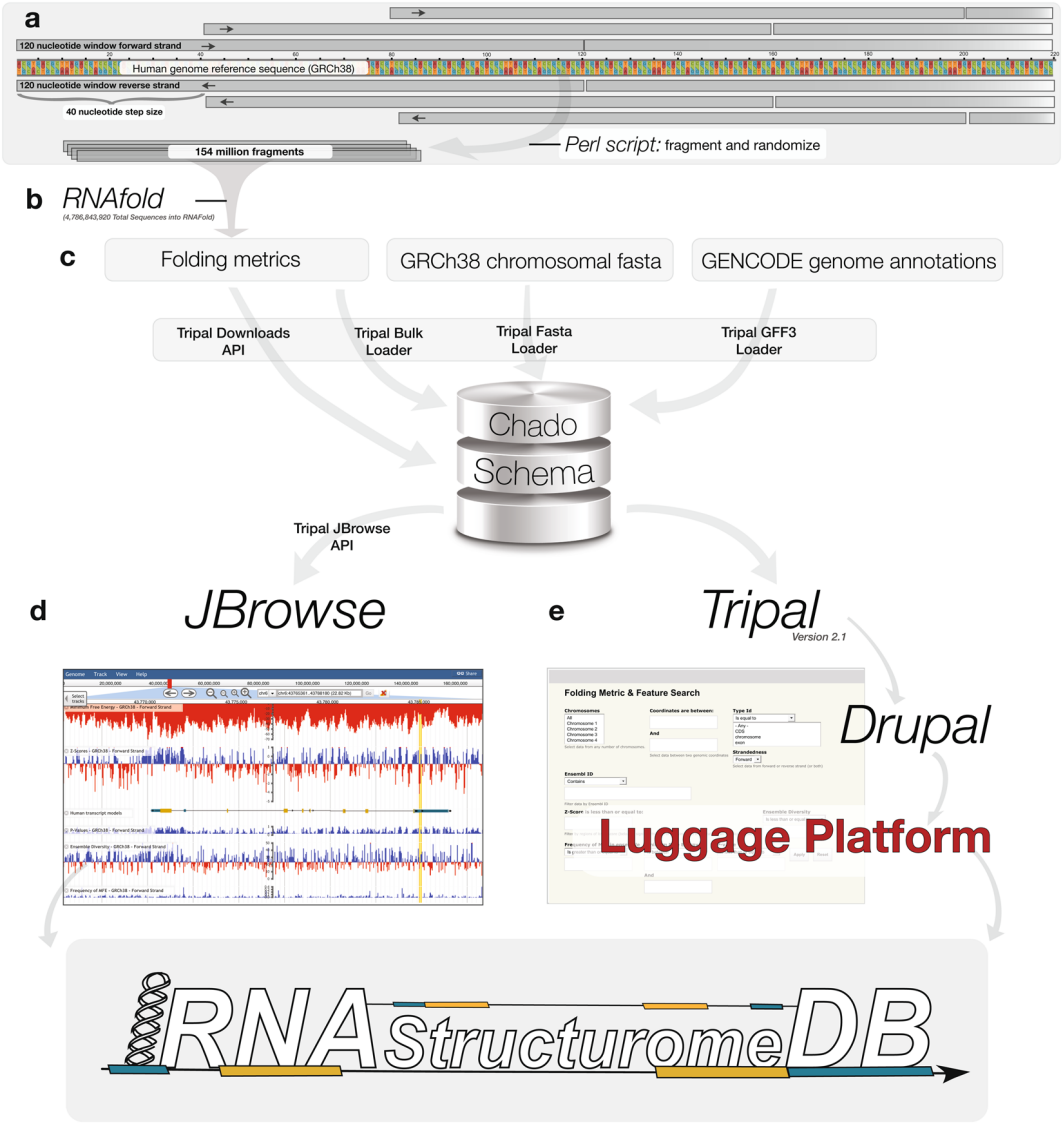
Overview of RNAStructuromeDB construction. **(a)** The human genome was fragmented into 154 million windows, with each scrambled thirty times using a Perl script. **(b)** This resulted in over 4.5 billion sequences to be folded in RNAfold and utilized in the creation of all folding metrics. **(c)** Folding metrics, chromosomal fasta files, and genomic annotations were all uploaded to the Chado schema using the Tripal API. Each set of data references the same underlying human genome sequence (hg38/GRCh38), a fact which allows Tripal to relate all data sets based on their respective coordinates. **(d)** This data (now related within the Chado schema) is pulled from the Chado schema on demand via the Tripal JBrowse API and used to generate tracks on the JBrowse genome browser. **(e)** In a separate process, Tripal is also able to populate tables for our data-table interface by acting as an interface between Chado and Drupal/Luggage.

Our database is housed on a Red Hat Enterprise Linux (RHEL7) server (provided by the Research IT group at Iowa State University http://researchit.las.iastate.edu) running Postgres 9.2, with all computational and genomic data stored within the Chado schema. Folding metrics were calculated on Iowa State University’s High Powered Compute cluster using RNAfold (version 2.2.10) and Perl (version 5). The website pages were built using Iowa State University’s Luggage platform (http://luggagedocs.info/), which is constructed on an underlying Drupal 7 framework. Tripal^99,100^ (version 2.1) was used to upload all data into the Chado schema and populate page “views” by later pulling relevant data from the Chado schema. JBrowse API (https://github.com/isubit/tripal_jbrowse_api) was used to generate JBrowse tracks directly from the Chado schema. An overview of this procedure is indicated in Fig. 4.

### Genomic sequence and annotations files

This analytical approach utilizes FASTA chromosomal DNA sequence files as input. For the human genome, the standardized reference sequence is generated by the Human Genome Reference Consortium. The latest major release of this human genome reference sequence (GRCh38/hg38) was acquired from Ensembl FTP server (ftp://ftp.ensembl.org/pub/release-90/fasta/homo_sapiens/dna/) as 24 separate chromosomal FASTA files (consisting of unmasked DNA sequence). Regions of sequence ambiguity and/or difficult to sequence nucleotides are depicted as the character “N” and were not considered in any calculations, but remain as placeholders within corresponding windows. Comprehensive Gencode genome annotations^54^ (release 26) were acquired from Gencode directly in the form of GFF3 files (ftp://ftp.sanger.ac.uk/pub/gencode/Gencode_human/release_26/gencode.v26.annotation.gff3.gz).

### RNA structure, partition function and z-score calculations

Each chromosomal FASTA file supplied the underlying sequence for metric calculations, and was analyzed every 40 nt using 120 nt windows. Each window fragment is run through RNAfold to calculate its MFE (value and fold) and partition function at 37 °C. To account for the reverse strand, each fragment is also converted to its reverse complement and run through the same process in a parallel script. Partition function calculations output an ensemble diversity score and an fMFE in the ensemble value for each window. Each “native” window sequence is then scrambled to produce 30 randomized versions which are run through RNAfold to calculate their MFE values as well. The MFE values of the native sequences are then compared to the MFE values of random sequences to generate a thermodynamic z-score, calculated with a method adapted from Clote *et al*.^66^ as illustrated in the following equation:

1 $$z-score=\frac{{\rm{\Delta }}{G}_{native}-\bar{\,{\rm{\Delta }}{G}_{random}}}{\sigma }$$

The randomization number was optimized to yield converged z-score values using the minimal randomization number. As a measure of z-score quality, we also calculate the fraction of MFE values that were lower than the native (the p-value) using Perl operations.

### Database web development

Web-based interfaces were prepared to put folding metric output into genomic context. An example of the pipeline used to generate the RNAStructuromeDB is shown in Fig. 4. Data tables were constructed by linking genome annotations to folding metrics based on genomic coordinates (whenever such relationships existed) and made available as searchable and downloadable CSV files; a preview of the data can be found alongside download links on the “Data Search” and “Gene Search” web pages, where users can view the data from individual windows and find links to view the corresponding MFE structures in forna^101^. Folding metric windows and Gencode annotations were also prepared as feature tracks for the JBrowse genome browsing interface; each folding metric window is displayed as a feature with directionality and each gene was visualized as “processed transcripts” (in order to visualize directionality, underlying exons, and UTRs when appropriate). Additionally, each folding metric (MFE, z-score, p-value, ED, and fMFE) was extracted and prepared as separate bigWig tracks for both forward and reverse strands – with each folding metric window trimmed to span only its initial 40 nt, allowing for proper visualization of each metric as a bar graph. We have left JBrowse customizable: users are given the option to upload tracks (in BAM, BED, bigWig, VCF, or FASTA formats) alongside RNAStructuromeDB metrics. Users are also able to download data from every track (whole sets or selected regions).

### MALAT1 Example

MALAT1 metrics were obtained from https://structurome.bb.iastate.edu/folding-metric-feature-search by filtering using Ensembl ID (ENSG00000251562.7) and downloading via the CSV link in the webpage. Statistical analyses were performed and graphs/tables were generated in Excel.

### VEGFA Example

VEGFA was navigated to at https://structurome.bb.iastate.edu/jbrowse/ by inputting its Ensembl ID (ENSG00000112715.21). All data was gathered from the JBrowse interface directly. All structures were generated using VARNA and annotated as described in Ray, *et al*.

### FNBP1 Example

A-to-I editing sites were obtained from ref.^88^ supplemental table five. Genomic coordinates were converted from human genome reference version hg18 to version hg38 using the UCSC reference conversion tool: https://genome.ucsc.edu/cgi-bin/hgLiftOver. Converted coordinates were then used to create bigWig tracks for each nucleotide site. These tracks were uploaded directly to JBrowse from the user interface for visualization along with folding metrics.

### Data availability

The datasets generated during the current study are available in the RNAStructuromeDB downloads repository, https://structurome.bb.iastate.edu/downloads or from corresponding author on reasonable request.

## Electronic supplementary material

Supplementary Information

Supplementary Table
