# Supplementary material for: RNAStructuromeDB: A genome-wide database for RNA structural inference
Source: Sci Rep. 2017 Dec 8;7:17269. doi: 10.1038/s41598-017-17510-y (PMC5722888; doi:10.1038/s41598-017-17510-y)
Supplement: Supplementary file 1 — Supplementary Information [file 41598_2017_17510_MOESM1_ESM.pdf]

Supplementary Information

RNAStructuromeDB: A genome-wide database for RNA structural inference

Ryan J. Andrews, Levi Baber, and Walter N. Moss*

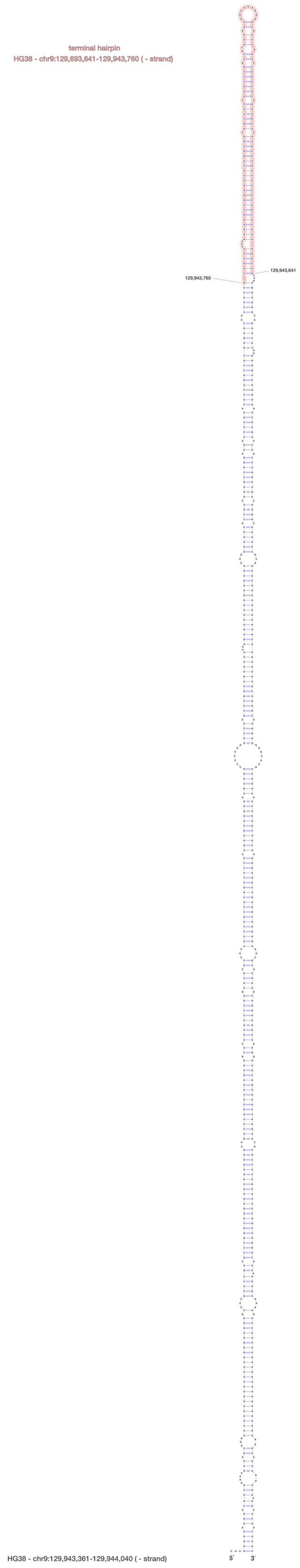

Supplementary Figure 1.
